# Alternative electrodes for HTMs and noble-metal-free perovskite solar cells: 2D MXenes electrodes†

**DOI:** 10.1039/c9ra06091j

**Published:** 2019-10-23

**Authors:** Junmei Cao, Fanning Meng, Liguo Gao, Shuzhang Yang, Yeling Yan, Ning Wang, Anmin Liu, Yanqiang Li, Tingli Ma

**Affiliations:** State Key Laboratory of Fine Chemicals, School of Petroleum and Chemical Engineering, Dalian University of Technology Panjin 124221 P. R. China liguo.gao@dlut.edu.cn; Graduate School of Life Science and Systems Engineering, Kyushu Institute of Technology Kitakyushu Fukuoka 808-0196 Japan tinglima@life.kyutech.ac.jp

## Abstract

The high cost of hole transporting materials (HTMs) and noble metal electrodes limits the application of perovskite solar cells (PSCs). Carbon materials have been commonly utilized for HTMs and noble-metal-free PSCs. In this paper, a more conductive 2D MXene material (Ti_3_C_2_), showing a similar energy level to carbon materials, has been used as a back electrode in HTMs and noble-metal-free PSCs for the first time. Seamless interfacial contact between the perovskite layer and Ti_3_C_2_ material was obtained using a simple hot-pressing method. After the adjustment of key parameters, the PSCs based on the Ti_3_C_2_ electrode show more stability and higher power conversion efficiencies (PCE) (13.83%, 27% higher than that (10.87%) of the PSCs based on carbon electrodes) due to the higher conductivity and seamless interfacial contact of the MXene electrode. Our work proposes a promising future application for MXene and also a good electrode candidate for HTM and the noble-metal-free PSCs.

## Abstract 2

The 2D Mxene material was successfully used as the counter electrode of the perovskite solar cell and achieved power conversion efficiencies of 13.84%.

## Graphical abstract

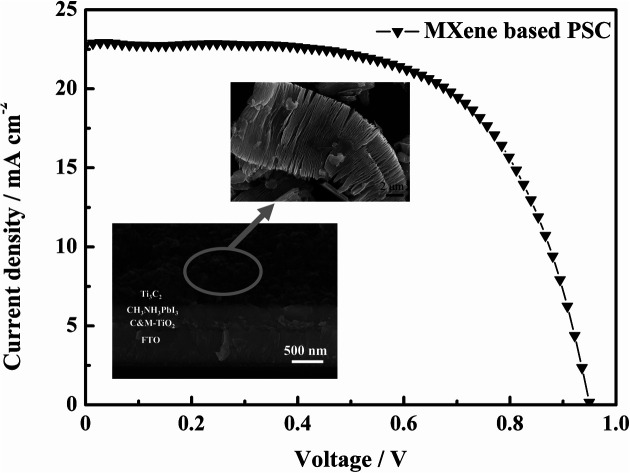

## Introduction

2D materials have excellent electronic, mechanical and optical properties, which have attracted much attention since the discovery of graphene monolayers in 2004.^1,2^ Recently, an additional 2D material, early transition metal carbide (Ti_3_C_2_), was synthesized by selectively etching the Al atoms in a layered hexagonal ternary carbide (Ti_3_AlC_2_) using aqueous hydrofluoric acid (HF).^3^ Ti_3_C_2_ is one of more than 70 different known transition metal carbides, carbonitrides and nitrides called MXenes. The general formula of MXenes is M_*n*+1_X_*n*_T_*x*_ (*n* = 1–3), where M is an early transition metal (such as Sc, Ti, Zr, Hf, V, Nb, Ta, Cr, Mo and others), X is C and/or N, and T_*x*_ stands for the surface terminations (hydroxyl, oxygen or fluorine).^4^ Various as-synthesized MXenes are endowed with excellent properties, such as good electronic conductivity,^5^ high hydrophilicity,^6^ highly transparent^7^ and convenient building blocks.^8^

The versatile chemistry of the MXenes allows the tuning of properties for applications including anti-friction properties,^9^ electromagnetic interference shielding,^10^ water purification,^11,12^ nanofiltration,^13^ dye adsorption,^14^ O_2_ evolution electrocatalysis,^15^ H_2_ generation,^16^ sensors,^17,18^ CO catalysis,^19^ N_2_ capture and NH_3_ production,^20^ antibacterial activity,^21^ electronics^22^ and lubrication.^23^ In our previous report, Ni_2_CO_3_(OH)_2_ nanosheets and/or nanoparticles were grown *in situ* on the surface of Ti_3_C_2_ to form a composite electrode, which possessed good supercapacitor properties.^24^

Organic and inorganic lead halide PSCs have shown remarkable development and prospective applications due to their unique advantages, such as high absorption coefficients, excellent carrier transport, low cost, tunable compositions, and excellent structures, thereby allowing simple fabrication by various processes.^25–28^ Although recent studies showed that the PCE of PSCs could achieve 23.7%,^29^ HTMs (Spiro-OMeTAD commonly used) and noble metal electrode (Au and Ag) materials are expensive and not beneficial to commercial applications. To solve this problem, researchers have developed HTM and noble-metal-free PSCs.^30,37^ In particular, the utilization of a carbon electrode in such devices has attracted a lot of attention.^31^ We used a super low-cost, coal-based carbon electrode in HTM and noble-metal-free PSCs and obtained a PCE of PSCs at 10.87%, where the PSCs fabricated with a coal-based carbon electrode exhibited more stability than conventional devices.^32^ However, the PCE of HTM and noble-metal-free PSCs is still lower than that of conventional devices. Due to its high conductivity and mobility, Ti_3_C_2_ materials provide a promising future for further improvements in the PCE of HTM and noble-metal-free PSCs. In our previous report, Ti_3_C_2_ was used as an additive incorporated into a perovskite absorber layer. A 2% enhancement in the device performance was achieved from the incorporation of a 0.03 wt% amount of Ti_3_C_2_, where Ti_3_C_2_ accelerated the charge transfer due to its high electrical conductivity and mobility.^33^ However, as a new emerging 2D material similar to graphene, the application of MXene in the field of PSCs is still short of intensive study.

In this paper, HTM and noble-metal-free PSCs have been fabricated with a 2D Ti_3_C_2_ electrode for the first time. A hot-pressing method was carried out for forming a seamless interfacial contact between the perovskite layer and Ti_3_C_2_ electrode. After optimizing the key parameters, the PCE of the champion device increased to 13.83%. Results show that the devices based on the Ti_3_C_2_ electrodes have good reproducibility and better stability than conventional devices. Our work proposes a promising future application for 2D MXenes and also a good candidate for HTM and noble-metal-free PSCs.

## Results and discussion

**Fig. 1:**
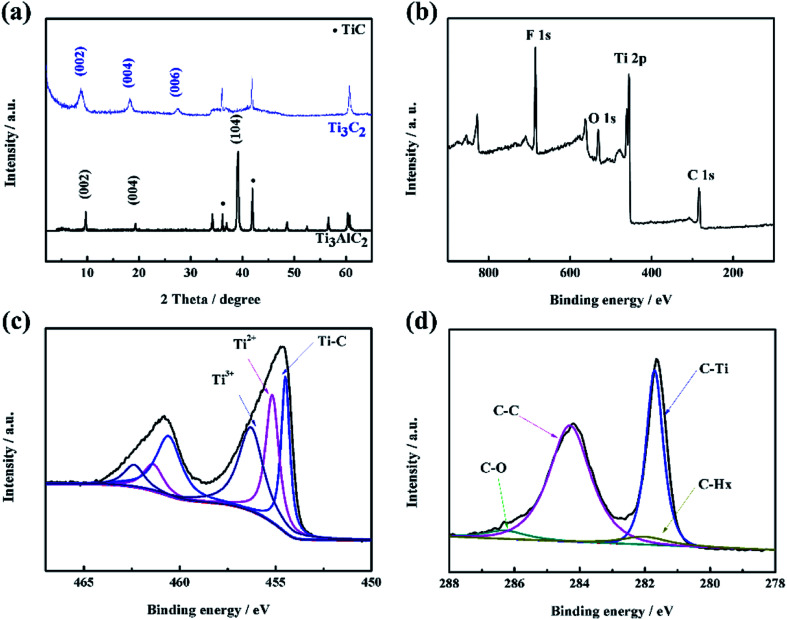
(a) XRD patterns of Ti_3_AlC_2_ and Ti_3_C_2_. (b) XPS spectra of the Ti_3_C_2_ sample, and the high-resolution spectra of (c) Ti 2p and (d) C 1s.

The Ti_3_C_2_ materials used in this work were prepared by etching Ti_3_AlC_2_ powders in HF solution to form a Ti_3_C_2_T_*x*_ structure. To illustrate the basic formation process, X-ray diffraction (XRD) and X-ray photoelectron spectroscopy (XPS) were used to characterize the materials, as shown in Fig. 1. The active layer (Al) can be selectively removed from the interlayers by disconnecting the metallic bonds between Al and Ti, resulting in 2D Ti_3_C_2_, as depicted in Fig. 1a. XRD patterns showed that the most notably intense peak at 2*θ* ≈ 39° of Ti_3_AlC_2_ disappeared. Standard peaks of the Ti_3_C_2_T_*x*_ structure appeared at (002), (004) and (006), where the (002) and (004) peaks were slightly shifted by a small degree due to the enlarged distance of the crystal face of Ti_3_C_2_T_*x*_ after HF etching.^30,34^ In order to further confirm the elemental distribution, XPS was conducted for Ti_3_C_2_T_*x*_. From the survey region (Fig. 1b), the signals belonging to the elements C, Ti, O and F were clearly detected without any signals for Al, which demonstrated that the Al element had been etched by HF acid, consistent with the XRD results. In the high-resolution XPS spectrum of Ti 2p (shown in Fig. 1c), the components centered at 454.6, 455.6, and 456.6 eV were assigned to Ti–C, Ti^2+^, and Ti^3+^, respectively. From the C 1s spectrum, the components fixed at 286.3, 284.3, 281.7, and 281.2 eV were assigned to C–O, C–C, C–H_*x*_, and C–Ti, respectively, which is in agreement with previous reports (Fig. 1d).^30^ Therefore, the 2D Ti_3_C_2_ materials were successfully obtained by HF etching the Ti_3_AlC_2_ powder.

**Fig. 2:**
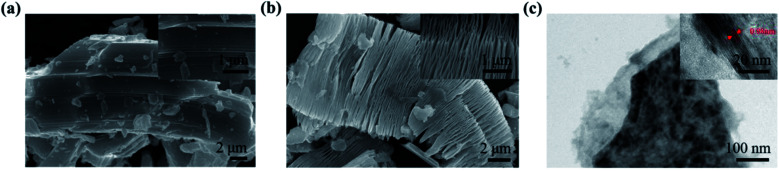
(a) SEM images of Ti_3_AlC_2_ powder before HF etching; the inset is an amplified image. (b) Ti_3_C_2_ exfoliation from HF acid; the inset is an amplified image. (c) TEM image of Ti_3_C_2_ nanosheets exhibiting a stack of multiple layers; the inset is an HRTEM image of the Ti_3_C_2_ nanosheets.

The morphology of Ti_3_AlC_2_ and Ti_3_C_2_ was characterized by a scanning electron microscope (SEM) and transmission electron microscope (TEM), as shown in Fig. 2. Ti_3_AlC_2_ consisted of a number of lamellar grains with densely aligned layered structures (Fig. 2a). After HF etching, exfoliation was achieved and the quasi-2D Ti_3_C_2_ sheets were obtained, as shown in Fig. 2b. The layers in Ti_3_C_2_ were clearly separated from each other in comparison to the unetched Ti_3_AlC_2_ powders, where the opened interspace was formed and the layered structure could be clearly observed. To further investigate the microstructure of the Ti_3_C_2_ nanosheets, TEM was tested as shown in Fig. 2c. The exfoliated 2D Ti_3_C_2_ nanosheets exhibited a stack of multiple layers. The high-resolution TEM (HRTEM) image (Fig. 2, inset) of the lattice fringe spacing between the two adjacent crystal planes of the nanosheets was determined to be 0.98 nm, which is consistent with the (002) crystal lattice in Fig. 1a.

**Fig. 3:**
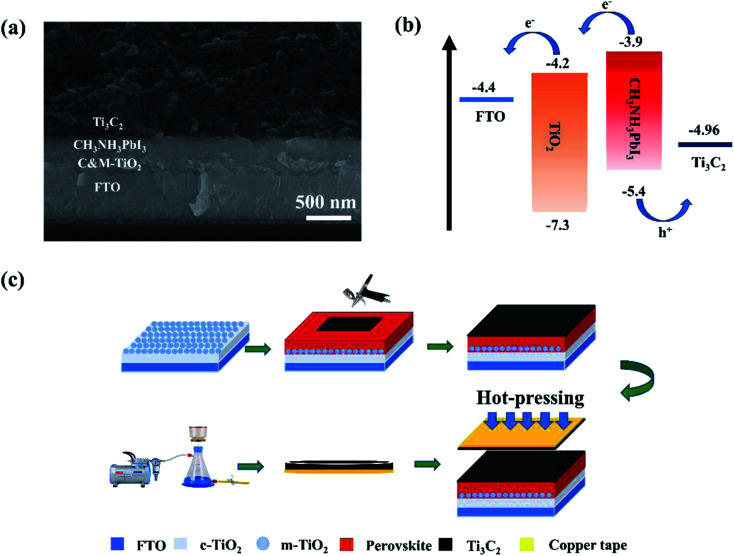
(a) Cross-sectional SEM image of the PSC based on Ti_3_C_2_ electrode. (b) Energy-level diagram of the PSC. (c) Schematic diagram of the fabrication process of Ti_3_C_2_ electrode by hot-pressing method.

The cross-sectional SEM image of this HTM and the noble-metal-free PSCs is shown in Fig. 3a, where the layers of FTO, compact TiO_2_ and mesoporous TiO_2_ (C&M TiO_2_), CH_3_NH_3_PbI_3_, and Ti_3_C_2_ electrode can be clearly differentiated. The Ti_3_C_2_ layer is in a seamless interfacial contact with the perovskite layer, which allows for holes to easily transfer from the light absorption layer to the Ti_3_C_2_ electrode. In order to improve the interfacial contact between the perovskite layer and the electrode materials, the Ti_3_C_2_ materials were ball-milled to form smaller particle sizes, resulting in the morphology change of Ti_3_C_2_. As shown in Fig. S1,† the interspaces between the Ti_3_C_2_ layers could not be easily distinguished. Ultraviolet photoelectron spectroscopy (UPS) was conducted to ensure a suitable energy level in Ti_3_C_2_ for the hole extraction, as shown in Fig. S2.† Results show that the work function of the Ti_3_C_2_ material is 4.96 eV, which matches well with the valence band of CH_3_NH_3_PbI_3_. The energy-level diagram of the Ti_3_C_2_ electrode-based device is shown in Fig. 3b, where both electrons and holes can be successfully extracted from the CH_3_NH_3_PbI_3_ layer. A schematic diagram of the device fabrication is shown in Fig. 3c, where two layers of Ti_3_C_2_ were fabricated. One was prepared by transferring the Ti_3_C_2_ electrode obtained from vacuum filtration to the copper tape. The other was prepared by spraying Ti_3_C_2_ paste onto the perovskite layer. To integrate these two Ti_3_C_2_ layers and induce a seamless interfacial contact at the perovskite layer/Ti_3_C_2_ electrode, the hot-pressing method was used at 85 °C and 0.4 MPa. Because of the thermoplasticity of polyvinyl acetate (PVAc), the prepared Ti_3_C_2_ electrode was tightly connected with the perovskite layer, and the two Ti_3_C_2_ layers made contact with each other without any interfacial crack, as shown in Fig. 3a.

**Fig. 4:**
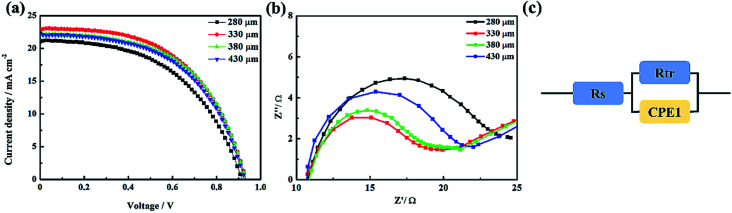
(a) *J*–*V* curves of devices with different thicknesses of the Ti_3_C_2_ electrode. (b) The Nyquist plot was measured under illumination (100 mW cm^−2^) at a bias voltage of 0.60 V. The frequency range is from 100 mHz to 1 MHz. (c) Equivalent circuit for fitting the Nyquist curve.

**Fig. 5:**
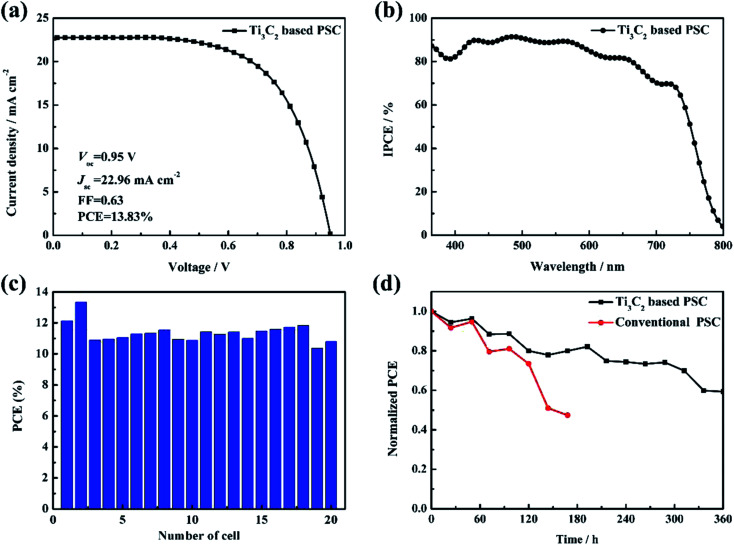
(a) *J*–*V* curves of the champion device based on the Ti_3_C_2_ electrode. (b) IPCE of the champion device. (c) PCE histogram of the PSCs obtained from the measurements of 20 devices. (d) Stability tests of the Ti_3_C_2_ electrode-based PSCs and conventional PSCs in an ambient atmosphere at room temperature (humidity 30%).

The key parameters and thickness of the Ti_3_C_2_ electrode were adjusted to achieve the optimal photovoltaic performance for the PSCs. The corresponding current density–voltage (*J*–*V*) characteristics of the PSCs are shown in Fig. 4a, and the photovoltaic properties are summarized in Fig. S3.† A four-point probe resistivity measurement system was employed to measure the square resistance of the Ti_3_C_2_ electrode, and the results are shown in Fig. S4.† When the thickness of the Ti_3_C_2_ electrodes increased from 280 μm to 330 μm, the square resistance decreased from 30.93 Ω □^−1^ to 25.34 Ω □^−1^. Thus, the open-circuit voltage (*V*_oc_), short-circuit current (*J*_sc_), and the fill factor (FF) of such devices all increased, as shown in Fig. 4a. To explain this phenomenon, a Nyquist plot was measured under illumination (100 mW cm^−2^) at a bias voltage of 0.60 V, which was measured at a frequency range from 100 mHz to 1 MHz (Fig. 4b). The equivalent circuit is presented in Fig. 5c. The series resistance (*R*_s_) is related to the external resistance, including wires and substrates, among others.^35,36^ In this work, PSCs based on the Ti_3_C_2_ electrodes were provided with similar *R*_s_ values (Fig. 4b). Under the premise of the same experimental conditions, except the thickness of the Ti_3_C_2_ electrode, the arc at high frequency is attributed to the charge transport resistance in the devices (*R*_tr_), which reflects the hole extraction and transport properties associated with the perovskite/Ti_3_C_2_ electrode interface. The decreased *R*_tr_ means that the hole could be extracted more efficiently as the thickness increased from 280 μm to 330 μm. However, a continuous increase in the thickness of the Ti_3_C_2_ electrodes would cause increasing resistance, and then induce a decline in the photovoltaic performance for the PSCs. It is presumed that when the thickness of the electrode is large, the transport distance and lifetime of the carrier are limited, resulting in a recombination of the electrons and holes. For further improvement in the performance of the PSC, a small amount of acetylene black was added to the Ti_3_C_2_ electrode. As shown in Fig. S5,† small acetylene black particles could fill the gaps of the large Ti_3_C_2_ particles for the holes to be extracted and transported efficiently. The optimal ratio of Ti_3_C_2_ to acetylene black was 5 : 1. The corresponding results are shown in Fig. S6 and S7.† In addition, PVAc as a binder played an important role in the performance of the devices. The optimal mass ratio of Ti_3_C_2_ to PVAc was 5 : 1, as well. The *J*–*V* curves and photovoltaic parameters of the devices with different ratios of Ti_3_C_2_ to PVAc are shown in Fig. S8 and S9.†

Fig. 5a depicts the photovoltaic performance of the champion device (FTO/C&M TiO_2_/perovskite/Ti_3_C_2_), where the highest PCE was 13.83% with a *V*_oc_ value of 0.95 V, *J*_sc_ value of 22.97 mA cm^−2^, and FF value of 0.63. The corresponding incident photon-to-electron conversion efficiency (IPCE) spectrum is shown in Fig. 5b. A high IPCE exceeding 80% was obtained in the wavelength window ranging from 400 nm to 750 nm, suggesting that most of the harvested photons in this range could be converted into electrical energy. One important aspect of PSC research and development is manufacturability (production capacity and yields) without large batch-to-batch variations. The results demonstrated that the fabrication of PSCs based on Ti_3_C_2_ electrodes has good reproducibility, as shown in Fig. 5c. The long-term stability results in Fig. 5d shows that the devices based on the Ti_3_C_2_ electrodes were more stable than conventional PSCs (FTO/C&M TiO_2_/Perovskite/Spiro-OMeTAD/Au), especially when the devices were stored at ambient atmosphere and room temperature (humidity 30%) without any encapsulation for 360 h. Evidently, the PSCs based on the Ti_3_C_2_ electrodes exhibited excellent stability, where Ti_3_C_2_ (about 300 μm) could act as an encapsulating layer, resulting in the isolation of air and water.

The PCE of the Ti_3_C_2_ electrode-based PSCs was lower than that of the conventional PSCs. To investigate the possible reason, photoluminescence (PL) spectra were obtained (Fig. S10†). The PL intensity of the perovskite covered with Ti_3_C_2_ film was higher than that of the perovskite coated with Spiro-OMeTAD and Au films, which demonstrates the significant non-radioactive recombination of most carriers at the perovskite/MXene electrode interface. This phenomenon, similar to the carbon electrode, could attribute to a bad contact at the perovskite/Ti_3_C_2_ electrode interface, which arises from the bulk physics properties of the Ti_3_C_2_ materials.^32^ However, the excellent electrical properties of Ti_3_C_2_ as an electrode material were compared with the coal-based carbon electrode. As shown in Fig. S11,† the photovoltaic performance of PSCs with the Ti_3_C_2_ electrode (denoted as Ti_3_C_2_-based PSCs) was significantly higher than that of PSCs with the coal-based carbon electrode (denoted as coal-based PSCs). This conclusion is because the Ti_3_C_2_ electrode exhibited better conductivity than the coal-based carbon electrode, as shown in Fig. S12.†

## Conclusions

We successfully fabricated seamless interfacial contact Ti_3_C_2_ electrodes for HTM and noble-metal-free PSCs through a hot-pressing method. Good reproducibility and better stability than conventional PSCs are demonstrated. After adjusting key parameters of the Ti_3_C_2_ electrode, the champion PCE (13.83%) of the PSCs based on the Ti_3_C_2_ electrode was obtained, which was much higher than that of the coal-based PSCs. This is because the square resistance of the Ti_3_C_2_ electrode was six times lower than that of the carbon electrode. Our work proposes a promising future application for MXene and also a good candidate for HTM and noble-metal-free electrodes for PSCs.

## Experimental section

### Device fabrication

1 g of Ti_3_AlC_2_ was slowly added to 30 mL of 40 %wt hydrofluoric acid and then stirred with a magnetic stir bar at 300 rpm for 24 h. The residue was washed with deionized water under centrifugation at 3500 rpm for 5 min. The precipitate was recovered while the supernatant was discarded, and this was repeated several times until the suspension pH reached a value of 6. The remaining sediment was collected and dried in the vacuum oven at 60 °C for 24 h. Then, the Ti_3_C_2_ sample was obtained. The obtained Ti_3_C_2_ particles were ball-milled for 48 h before use. For the Ti_3_C_2_ electrodes, two layers of Ti_3_C_2_ were prepared. One was prepared by transferring the Ti_3_C_2_ film obtained from vacuum filtration to the copper tape. The other was prepared by spraying Ti_3_C_2_ paste onto the perovskite layer. The optimized components in the Ti_3_C_2_ paste were 0.03 g MXene powder, 0.006 g acetylene black, and 0.072 g PVAc in each milliliter of isopropanol solvent, where PVAc in the paste acted as a binder. The Ti_3_C_2_ paste was sprayed onto the as-prepared photoanode, which was placed on a 90 °C hot plate. Then, the Ti_3_C_2_ film was hot-pressed on the perovskite at 85 °C under 0.4 MPa pressure for 15 s. Details of the preparation process for the devices are described in ESI.†

## Conflicts of interest

There are no conflicts to declare.

## Supplementary Material

RA-009-C9RA06091J-s001
