# Supplementary material for: Alternative electrodes for HTMs and noble-metal-free perovskite solar cells: 2D MXenes electrodes
Source: RSC Adv. 2019 Oct 23;9(59):34152–7. doi: 10.1039/c9ra06091j (PMC9073695; doi:10.1039/c9ra06091j)
Supplement: RA-009-C9RA06091J-s001 [file RA-009-C9RA06091J-s001.pdf]

Electronic Supplementary Material (ESI) for RSC Advances.
This journal is © The Royal Society of Chemistry 2019

Supporting Information

Alternative Electrodes for HTM and Noble-metal-free Perovskite Solar Cells: 2D MXene Electrode

Junmei Cao,[1] Fanning Meng,[1] Liguo Gao,[1,*] Yeling Yan,[1] Ning Wang,[1] Anmin Liu,[1] Yanqiang Li,[1] Tingli Ma,[2,*]

[1] *State Key Laboratory of Fine Chemicals, School of Petroleum and Chemical Engineering, Dalian University of Technology, Panjin, 124221, P. R.China.*

[2] *Graduate School of Life Science and Systems Engineering, Kyushu Institute of Technology, Kitakyushu, Fukuoka 808-0196, Japan.*

**Corresponding author: liguo.gao@dlut.edu.cn; tinglima@life.kyutech.ac.jp.*

1 Experimental Section

1.1 Materials

Fluorine-doped tin oxide (FTO, 15 Ω/□) was purchased from Yingkou OPV Tech New Energy Co., Ltd. Tetrabutyl titanate (97%), diethanolamine (99.5%), dimethyl sulfoxide (99.9%), isopropanol (IPA) (99.9%) and PbI_2 (99%) were all purchased from Sigma-Aldrich, respectively. TiO_2 paste was purchased from Dyesol (30 NRD). N,N-dimethylformamide (99.9%, superdry) was purchased from J&K Scientific Ltd. Methylamine iodine (MAI) (99.9%) and Spiro-OMeTAD were purchased from Xi'an Polymer Light Technology Corp. Au nanowire (99.9%) was purchased from Alfa Aesar. Polyvinyl acetate (PVAc) was purchased from Xiya chemical industry Co., Ltd. Ti_3AlC_2 powder was purchased from Luoyang Tongrun Info Technology Co., Ltd. And acetylene black (particle size, 30-40 nm) was purchased from Denka.

1.2 Fabrication of photoanodes

Compact TiO_2 layer (C-TiO_2) was deposited on the FTO substrates by spin-coating TiO_2 organic sol and sintered in a furnace at 450°C. A commercial TiO_2 paste diluted in ethanol was spin coated on C-TiO_2 to form mesoporous TiO_2 layer (M-TiO_2). PbI_2 dissolved in DMF was spin coated onto the FTO/C-TiO_2/M-TiO_2 substrate at 3000 rpm for 30 s. Subsequently, MAI dissolved in IPA was spin coated at 5,000 rpm for 30 s, annealing at 125 °C for 20 min.

1.3 Fabrication of PSCs

1 g of Ti_3AlC_2 was slowly added to 30 ml of 40%wt hydrofluoric acid and then stirred with a magnetic stir bar at 300 rpm for 24 h. The residue was washed with deionized water under the centrifugation at 3500 rpm for 5 min. The precipitate was recovered while the supernatant was discarded, and this was repeated for several times until the suspension pH reached to 6. The remaining sediment was collected and dried in the vacuum oven at 60 °C for 24 h. Then, Ti_3C_2

sample was obtained. The obtained Ti_3C_2 particles were ball-milled for 48 h before use. For Ti_3C_2 electrodes, two layers of Ti_3C_2 were prepared. One was prepared by transferring Ti_3C_2 film obtained from vacuum filtration to copper tape. The other was prepared by spraying Ti_3C_2 paste on the perovskite layer. The components in Ti_3C_2 paste are 0.03 g Ti_3C_2 powder, 0.006 g acetylene black, and 0.072 g PVAc in each milliliter of isopropanol solvent. The PVAc in the paste acts as a binder. The Ti_3C_2 paste was spraying on the as-prepared photoanode, which was placed on 90 °C hot plate. Then, the Ti_3C_2 film was hot-pressed on it at 85 ℃ and 0.4 Mpa pressure for 15 s.

1.4 Device characterization

Surface and cross-sectional morphologies of both Ti_3C_2 material and devices based on Ti_3C_2 electrodes were observed by a field-emission scanning electron microscopy (SEM, FEI Nova NanoSEM 450). The structure properties of Ti_3C_2 material were performed by X-ray diffraction (XRD) analysis (XRD-7000S, Shimadzu) from 5° to 80°, Transmission electron microscopy (TEM, Philips Tecnai G2 F30 S-TWIN), and X-ray photoelectron spectroscopy (XPS, Thermo Scientific, ESCLAB 250Xi). The work function of Ti_3C_2 material was measured by ultraviolet photoelectron spectroscopy (UPS, ESCALAB™250Xi), using a He I irradiation source (hv = 21.2 eV).

The cells were irradiated by Peccell-L15 solar simulator (Peccell, Japan) under AM 1.5G 100 mW/cm^2. Keithley 2460 (Keithley, America) was used to record the J-V characteristics of the cells. The test condition was that active area (0.08 cm^2) was defined by a black mask, where scan rate was 0.2 V/s for both reverse and forward scan. The resistance of the Ti_3C_2 electrodes was measured by a four-point probe resistivity measurement system (Four Probes Tech, Guangzhou, China, RTS-9). Nyquist plot measurements were conducted with an electrochemical

workstation (Zennium Zahner, Germany) under light illumination, under a 0.6 V forward bias voltages and with 20 mV amplitude of AC perturbation ranging from 100 mHz to 1 MHz. The incident photon to current conversion efficiency (IPCE) was performed employing a xenon lamp coupled with a monochromator (PEC-S20, Peccell) controlled by a computer from 365 nm to 1020nm. The photoluminescence (PL) was conducted using an optically triggered streak camera system (C5410, Hamamatsu). All samples were photo-excited using a 517 nm laser with a repetition rate of 76 MHz (Mira900, Coherent). Each of the samples was tested 3-5 times, and the average data were obtained.

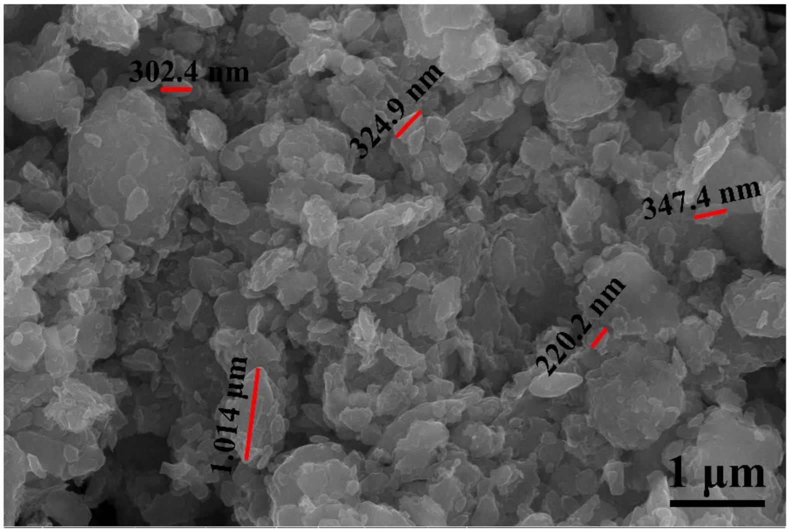

Figure S1. SEM images and size of Ti_3C_2 after ball milling

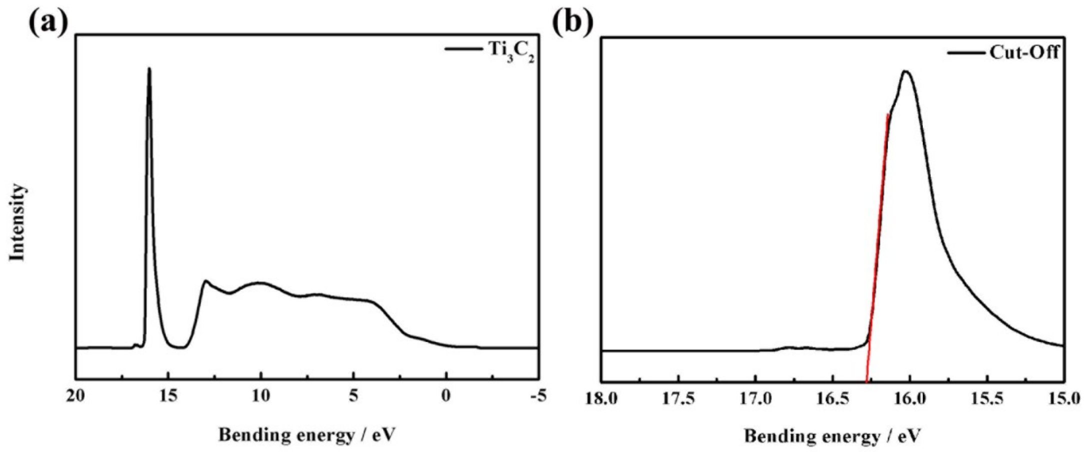

Figure S2. (a) The full UPS spectrum of Ti_3C_2. (b) Secondary-electron cut o□ for work function (WF) determination, where WF is 4.96 eV (21.22 eV - 16.26 eV).

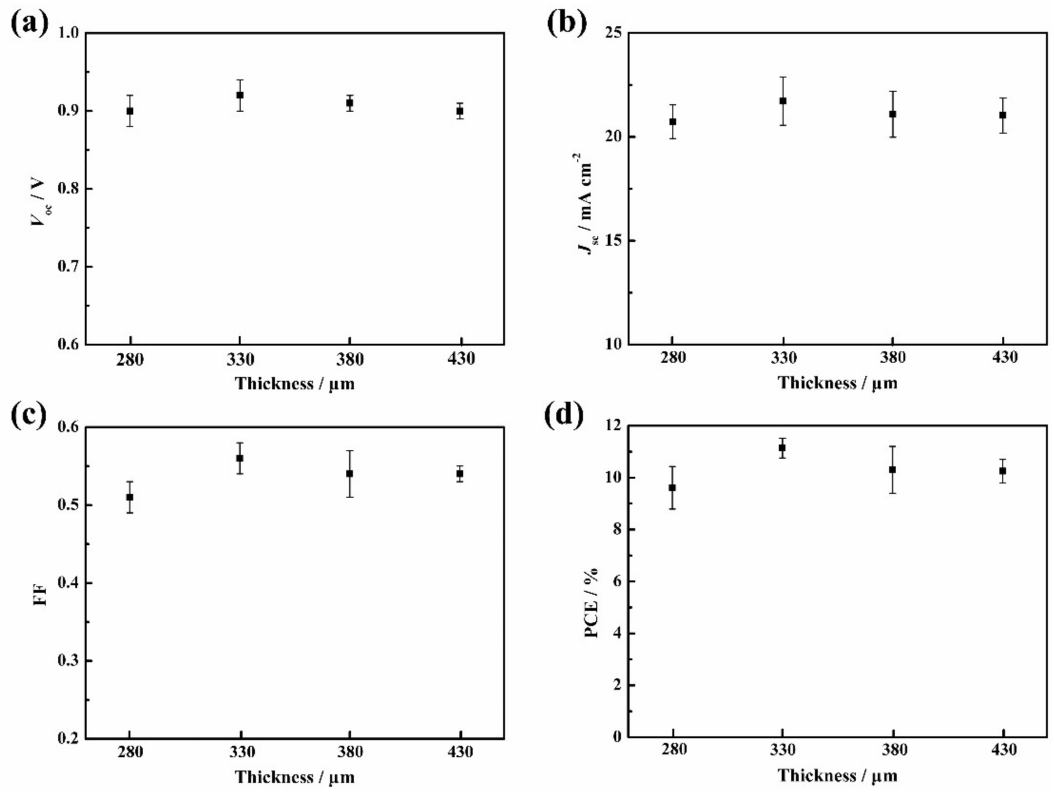

Figure S3. Photovoltaic parameters of PSCs based on Ti_3C_2 electrode with different thickness.

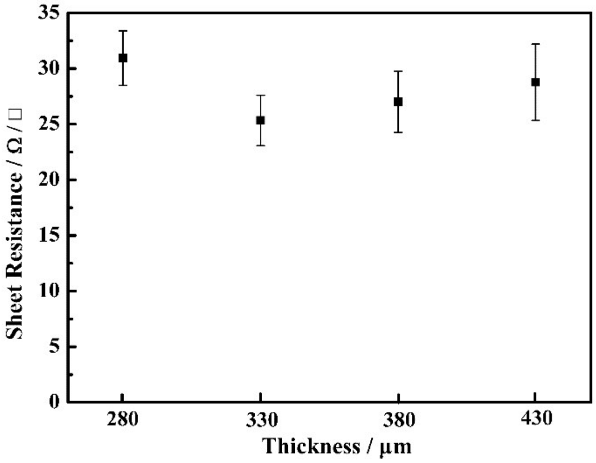

Figure S4. The square resistance of Ti_3C_2 electrodes with different thickness.

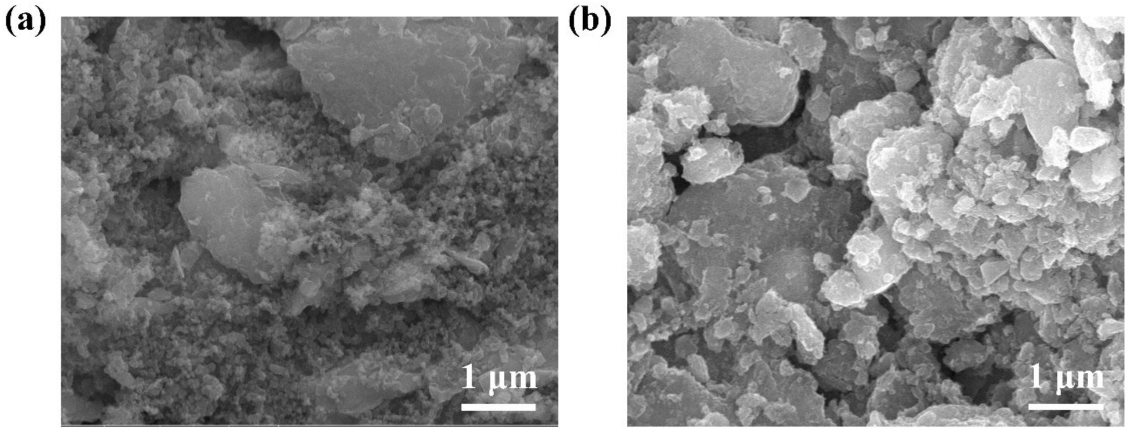

Figure S5. SEM images of Ti_3C_2 electrode with (a) and without (b) acetylene black at a given ratio of PVAc.

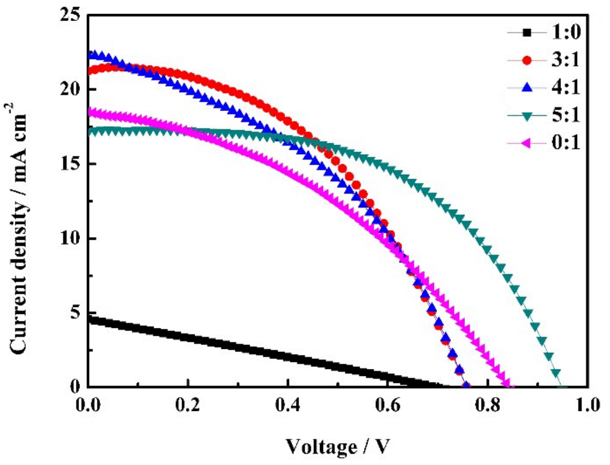

Figure S6. J-V curves of devices based on Ti_3C_2 electrodes with different ratios of Ti_3C_2 to acetylene black. (The ratios of Ti_3C_2 to acetylene black are 1:0, 3:1, 4:1, 5:1, and 0:1, respectively.)

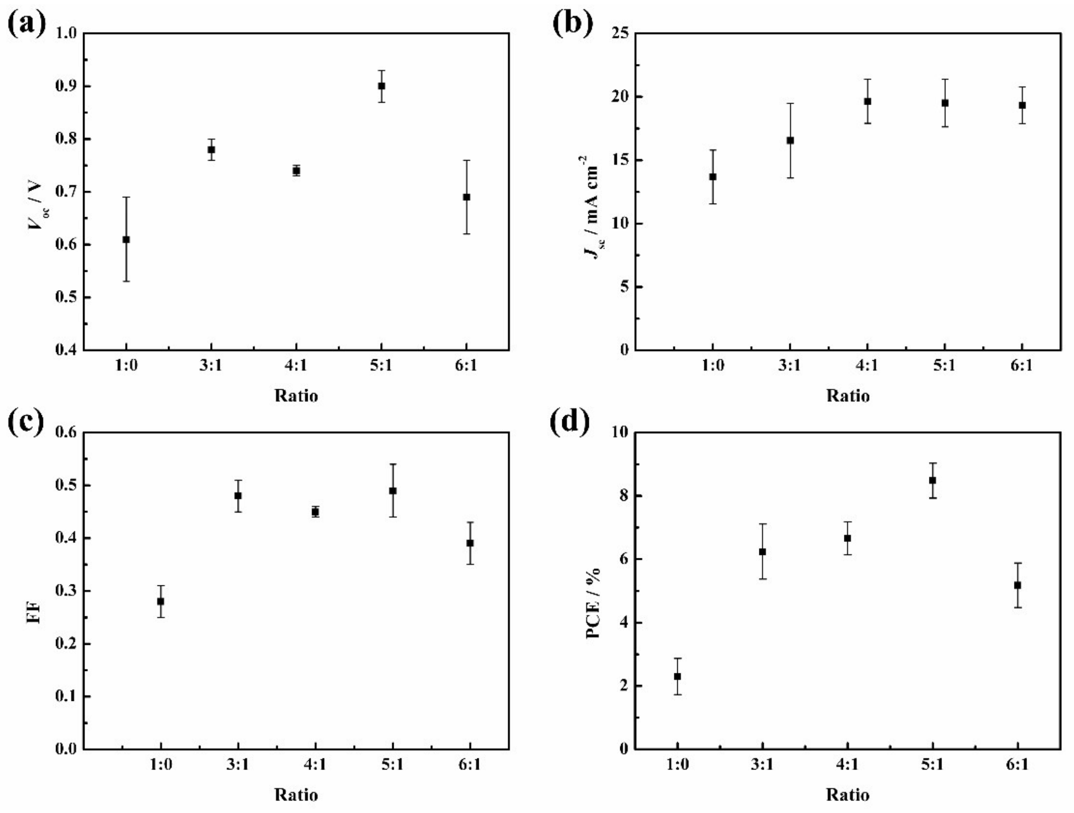

Figure S7. Photovoltaic parameters of PSCs based on Ti_3C_2 electrode with different ratios of Ti_3C_2 to acetylene black. (The ratios of Ti_3C_2 to acetylene black are 1:0, 3:1, 4:1, 5:1, and 0:1, respectively.)

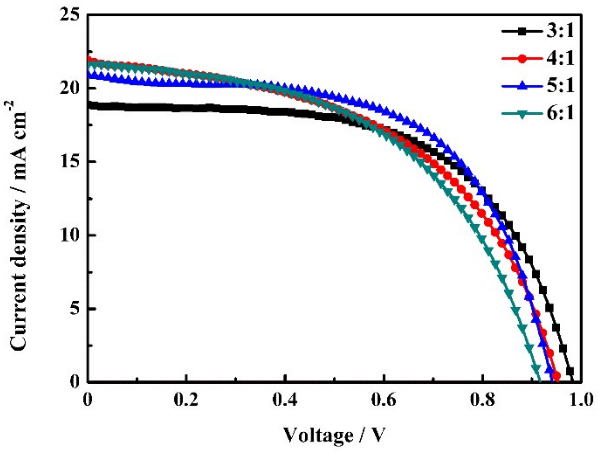

Figure S8. J-V curves of PSCs based on Ti_3C_2 electrode with different ratios of Ti_3C_2 to PVAc, where the ratios of Ti_3C_2 to acetylene black is 5:1. (The ratios of Ti_3C_2 to PVAc are 3:1, 4:1, 5:1, and 6:1, respectively.)

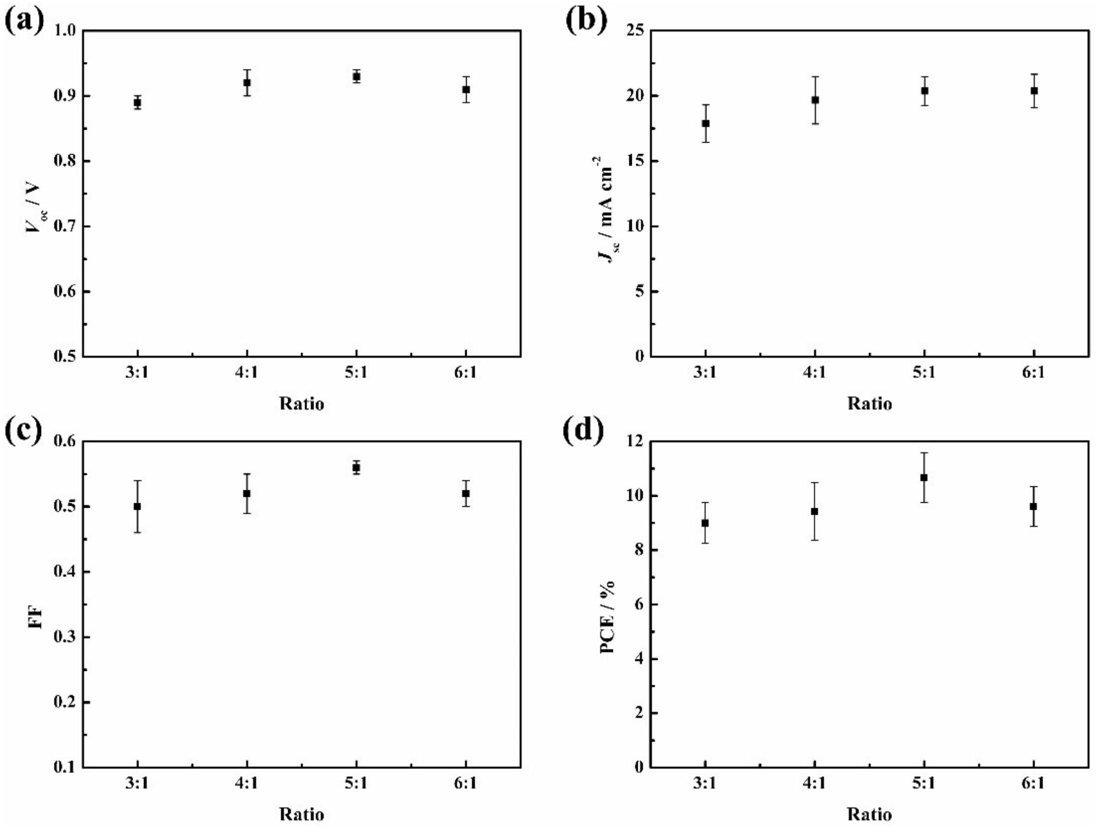

Figure S9. Photovoltaic parameters of PSCs based on Ti_3C_2 electrode with different ratios of Ti_3C_2 to PVAc. (The ratios of Ti_3C_2 to PVAc are 3:1, 4:1, 5:1, and 6:1, respectively.)

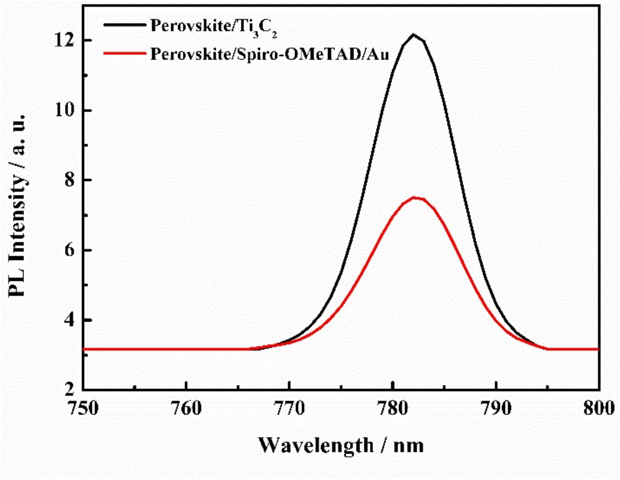

Figure S10. PL spectra of perovskite/ Ti_3C_2 and perovskite/Spiro-OMeTAD/Au samples.

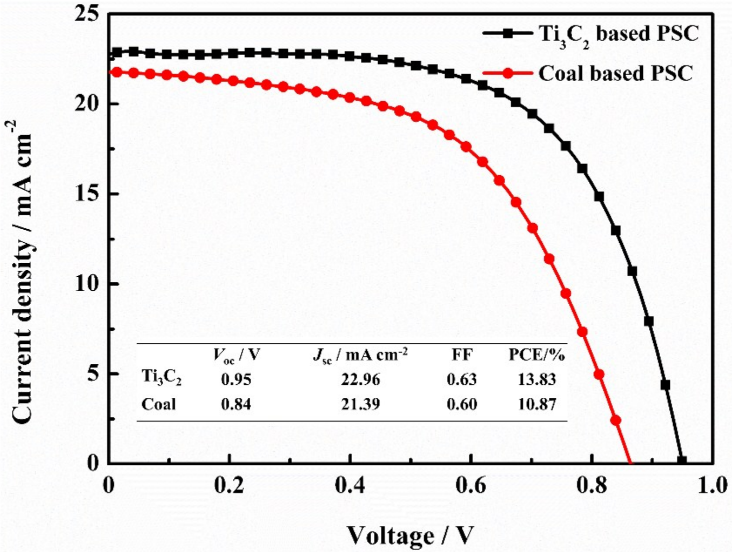

p///

Figure S11. J-V curves of Ti_3C_2 based PSC and coal based PSC.

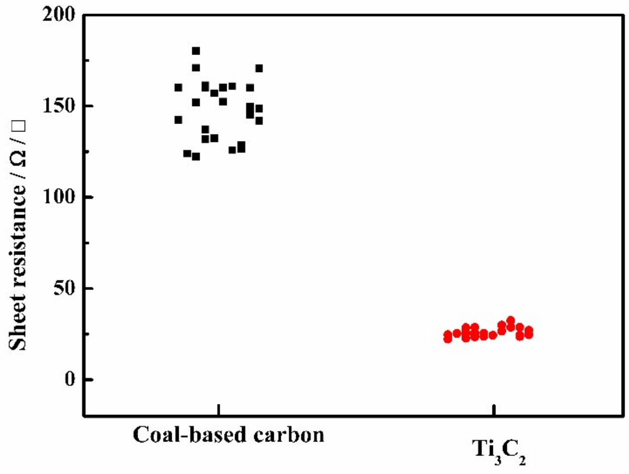

Figure S12. The square resistance of Ti_3C_2 electrodes and coal-based carbon electrodes at the same thickness.
